# IgG4-Related Sclerosing Cholangitis: Rarely Diagnosed, but not a Rare Disease

**DOI:** 10.1155/2021/1959832

**Published:** 2021-12-21

**Authors:** Sylvia Drazilova, Eduard Veseliny, Patricia Denisa Lenartova, Dagmar Drazilova, Jakub Gazda, Ivica Grgurevic, Martin Janicko, Peter Jarcuska

**Affiliations:** ^1^2^nd^ Department of Internal Medicine, PJ Safarik University in Kosice and L. Pasteur University Hospital, Trieda SNP 1, 040 11 Kosice, Slovakia; ^2^Department of Infectology and Travel Medicine, PJ Safarik University in Kosice and L. Pasteur University Hospital, Rastislavova 43, 040 01 Kosice, Slovakia; ^3^1^st^ Faculty of Medicine, Charles University, Katerinska 1660/32, 121 08 Nove Mesto, Prague, Czech Republic; ^4^Department of Gastroenterology, Hepatology and Clinical Nutrition, University of Zagreb School of Medicine and University Hospital Dubrava, Avenija Gojka Suska 6, 10000 Zagreb, Croatia

## Abstract

IgG4-related sclerosing cholangitis, a biliary manifestation of an IgG4-related disease, belongs to the spectrum of sclerosing cholangiopathies which result in biliary stenosis. It presents with signs of cholestasis and during differential diagnosis it should be distinguished from cholangiocarcinoma or from other forms of sclerosing cholangitis (primary and secondary sclerosing cholangitis). Despite increasing information and recently established diagnostic criteria, IgG4-related sclerosing cholangitis remains underdiagnosed in routine clinical practice. The diagnosis is based on a combination of the clinical picture, laboratory parameters, histological findings, and a cholangiogram. Increased serum IgG4 levels are nonspecific but are indeed a part of the diagnostic criteria proposed by the Japan Biliary Association and the HISORt criteria for IgG4-SC. High serum IgG4 retains clinical utility depending on the magnitude of elevation. Approximately 90% of patients have concomitant autoimmune pancreatitis, while 10% present with isolated biliary involvement only. About 26% of patients have other organ involvement, such as IgG4-related dacryoadenitis/sialadenitis, IgG4-related retroperitoneal fibrosis, or IgG4-related renal lesions. A full-blown histological finding characterized by IgG4-enriched lymphoplasmacytic infiltrates, obliterative phlebitis, and storiform fibrosis is difficult to capture in practice because of its subepithelial localization. However, the histological yield is increased by immunohistochemistry, with evidence of IgG4-positive plasma cells. Based on a cholangiogram, IgG-4 related sclerosing cholangitis is classified into four subtypes according to the localization of stenoses. The first-line treatment is corticosteroids. The aim of the initial treatment is to induce clinical and laboratory remission and cholangiogram normalization. Even though 30% of patients have a recurrent course, in the literature data, there is no consensus on chronic immunosuppressive maintenance therapy. The disease has a good prognosis when diagnosed early.

## 1. Definition

IgG4-related sclerosing cholangitis (IgG4-SC) is a biliary manifestation of IgG4-related disease (IgG4-RD). IgG4-RD is an immune-mediated fibroinflammatory disease that can affect almost any organ. The disease may present either as diffuse fibroinflammation or as the formation of inflammatory pseudotumors in the affected organ. Typical histological signs for IgG4 disease are IgG4-enriched lymphoplasmacytic infiltrate, obliterative phlebitis, storiform fibrosis, and variable presence of eosinophils in affected organs [1–3]. Most patients have elevated serum IgG4 levels. A good initial response to corticoids is characteristic as well [4]. In IgG4-SC, lymphoplasmacytic inflammation affects the bile duct wall; however, it usually presents with other organ manifestations, especially with autoimmune pancreatitis [5, 6]. The term IgG4-associated sclerosing cholangitis was replaced with the formal name “IgG4-related sclerosing cholangitis” at the 1st International Symposium on IgG4-related disease [7]. This nomenclature aims to emphasize not only the similarities with primary sclerosing cholangitis (PSC) but also the strong need to distinguish between the two diseases, as they have diametrically different treatment and prognoses.

## 2. Epidemiology

There are only limited epidemiological data on IgG4-SC, some of which evaluate the occurrence of IgG4-SC only indirectly. There are several reasons for this. IgG4-RD is a new disease, whose diagnostic criteria have only recently been accepted worldwide. This has raised global awareness of the disease, but the disease does not have its own unique International Statistical Classification of Diseases and Related Health Problems (ICD-10) code, which complicates epidemiological studies. The concept of IgG4 disease was preceded by information about its individual organ manifestations. The most studied one is autoimmune pancreatitis (AIP), which IgG4-SC is typically associated with. Most literature data come from Japan. The first mention of AIP dates back to 1995; however, the diagnostic criteria for AIP were first proposed by the Japan Pancreas Society in 2006 [8, 9]. In the same year, the new clinicopathological entity of IgG4-SC associated with autoimmune pancreatitis was proposed by Kamisawa et al. [10]. Epidemiological studies focusing on IgG4-SC's prevalence and incidence have many limitations:

1. IgG4-RD is a relatively new disease entity with unification of a host of previous clinical entities under the umbrella term “IgG4-RD” only achieved in 2012 [4]
2. The diagnostic criteria for IgG4-SC have not yet been unified, with the HISORt and Japan Biliary Association criteria sharing many similarities but also some important differences [2, 11]
3. There is controversy around whether type 1 IgG4-SC should be considered IgG4-SC or simply AIP with associated biliary sclerosis

Another factor that affects epidemiological studies is the interpretation of type 1 IgG4-SC, which is associated with a lower rate of bile duct stenosis and is mostly associated with the AIP. One group of experts does not approve of this type, because it believes that such stenosis could be caused by external compression of the intrapancreatic portion of the bile duct by the inflamed pancreatic tissue and not by inflammation of the bile duct [12].

The other group of experts presumes that type 1 IgG4-SC may occur even without concurrent AIP, which is supported by case series, large series, and even histological findings [3, 13–16].

Exclusion of type 1 IgG4-SC would have a significant impact on epidemiological data, because type 1 IgG4-SC occurs most frequently and according to Tanaka makes up to 64% of all IgG4-SC [17].

These studies have several limitations, and it is generally assumed that they underestimate the real incidence and prevalence. In 2011, Kanno et al. conducted the Nationwide Epidemiological Survey of Autoimmune Pancreatitis in Japan with the following results: the overall prevalence rate of AIP was 4.6 cases per 100,000 inhabitants and the annual incidence rate was 1.4 per 100,000 inhabitants [18]. Based on this information and the fact that IgG4-SC is present in about 40% of patients with AIP, we can indirectly conclude that the incidence of IgG4-SC is approximately 0.5 new cases per 100,000 inhabitants and the prevalence is 1.8 cases per 100,000 inhabitants [19]. On the other hand, approximately 10% of patients with IgG4-SC are not diagnosed with AIP; therefore, the estimated prevalence of IgG4-SC could theoretically be as high as 2.0 cases per 100,000 inhabitants. Finally, in 2020, Tanaka et al. published the first epidemiological study evaluating 1,045 IgG4-SC patients from 532 centers in Japan and showed that the prevalence of IgG4-SC in 2018 was 2.18 (95% confidence interval, 2.13–2.23) per 100,000 inhabitants, which is comparable to other data [20]. Thus, we can conclude that the prevalence of IgG4-SC in Japan is higher than the prevalence of PSC, which in 2016 was 1.80 (95% CI, 1.75–1.85) [21].

An indirect estimate of the prevalence of IgG4-SC can be obtained from case series data on the percentage of IgG4-RD cases with biliary involvement. In 2015, Inoue et al. analyzed a cohort of 235 patients from 8 Japanese IgG4-RD centers, 64% of whom had histologically verified disease. Pancreatic involvement was present in 60% and bile duct involvement in 13% of them [22]. On the other hand, a study published by Chinese authors reports 118 patients with IgG4-RD, 38% of whom had AIP and in 17.8% biliary tree involvement was present [23]. The findings of another Chinese study are similar, where, among 200 patients with IgG4-RD, pancreatic and biliary tract involvement was seen in 38.5% and 19% of patients, respectively [24]. Even lower prevalence of these organ manifestations was observed in a study by Wallace et al. from USA from 2015, which included 125 patients with histologically verified IgG4-RD. Only 19.2% of patients had pancreatic involvement and only 9.6% had biliary tract involvement [25]. If we want to indirectly judge the epidemiology of IgG4-SC in Europe, three works are available. The first included 41 patients with IgG4-RD from Italy. Autoimmune pancreatitis was the most common organ manifestation in 41% of patients, with the biliary tree being affected in 9.8% of patients [26]. The second is from Spain and evaluates 55 patients with IgG4-RD diagnosed in 12 Spanish hospitals. In this study, 16% of patients had pancreas involvement and only 4% had bile duct involvement. The smallest is the study from France, which included 25 patients with IgG4-RD, up to 52% with AIP and up to 32% with IgG4-SC [27], while 10% presented with isolated biliary involvement. The prevalence variability is mainly due to sampling error from these small sample sizes and referral bias and perhaps due to differences in clinical manifestation in different patient populations (e.g., perhaps due to the differences in HLA serotypes mentioned in the section on pathogenesis). The limited sample size of European studies also prevents the generalization of epidemiological data.

IgG4-SC is a disease of elderly patients. Most patients are diagnosed in the 6th-7th decade; the disease has not yet been described in children and adolescents [28, 29]. Only 0.7% of patients had the disease diagnosed in the second decade of life [20]. The median age at diagnosis in a different case series is comparable and represents 66–67 years in Japan, 62 years in USA, and 61 years in the UK [11, 20, 29, 30]. By contrast, patients with PSC are younger, and their mean age at diagnosis varies in the Western population from 35 to 47 years [31]. Both IgG4-SC and PSC predominantly affect men. Male patients represent 74% to 85% of all IgG4-SC patients. If IgG4-SC occurs in women, the IgG4-SC without AIP is more common [13].

## 3. Pathogenesis of IgG4-SC

IgG4-SC is a disease of unknown etiology with a multifactorial pathogenesis. IgG4-RD typically presents with polyclonal hypergammaglobulinemia and elevated serum IgG4 levels [32]. Some authors consider IgG4-SC to be an autoimmune disease because some patients have antinuclear antibodies and respond well to corticosteroid treatment or to rituximab [9, 33, 34]. The theory of an autoimmune basis of IgG4-RD is supported by the identification of several autoantigens (galectin-3, laminin 111, and annexin A 11). IgG4 galectin-3 autoantibodies are present in a portion of patients with IgG4-RD and correlate with galectin-3 plasma levels. Anti-laminin-511 E-8 IgG autoantibodies are targeted against laminin 511 in approximately half of patients with AIP. IgG1-mediated proinflammatory autoreactivity against annexin A11 in patients with IgG4-RD may be attenuated by formation of annexin A11-specific IgG4 antibodies in IgG4-SC and AIP patients [35–37].

IgG4 physiologically makes up to 3–6% of the total amount of IgG [28]. We do not know whether IgG4 has a proinflammatory or anti-inflammatory character; evidence suggests that IgG4 has an anti-inflammatory role in allergy, is pathogenic in certain autoimmune conditions (e.g., pemphigus), and supports an immune-tolerant state in helminthic infections [38, 39]. However, IgG4 appears to be a neutralizing antibody that fails to secure a complement, has weak binding to the Fc receptor, and is unable to form large immunocomplexes [38, 40, 41]. Some authors believe that excessive IgG4 production occurs secondarily, with the aim of attenuating the extensive immune response in IgG4-RD. This theory is supported by the fact that IgG4 interacts with the Fc portion of IgG in a way that mimics the rheumatoid factor [42]. In contrast to other autoimmune diseases, men are more commonly affected (80%) with higher mean age [43]. A theory that IgG4-SC should not be considered an autoimmune disease is supported by the finding of increased levels of regulatory T cells (Tregs) in IgG4-SC, whereas, in patients with classic autoimmune diseases, Tregs are reduced [44, 45]. Concerning the “allergic fibrosis theory,” the current prevalent approach states that Th2 is prevalent in IG4-RD patients with allergic disease [46]. Most IgG4-RD patients are not atopic, though most of them have eosinophilia and higher levels of IgE in the peripheral blood. These findings may imply that processes inherent to IgG4-RD itself rather than atopy per se contribute to the eosinophilia and IgE elevation [47].

Physiologically, IgG4 is formed as a result of strong or repeated antigen stimulation to induce tolerance [28]. Elevated IgG4 levels may be a protective mechanism for long-term antigen exposure [48]. Furthermore, some authors consider IgG4-SC to be a lymphoproliferative disease [43]; but current consensus does not believe this to be the etiology and no conclusive data is available for any of these possibilities (including lymphoproliferative disorder).

Genetic predisposition plays an important role in the development of IgG4-SC. HLA serotypes DRB1∗0405 and DQB1∗0401 are more common in IgG4-SC in the Japanese population, though not in other ethnic groups [49, 50]. In an English multicentric study, HLA-DRB1∗0301-DQB1∗0201 was found to be more common in patients with IgG4-SC and IgG4-AIP [51]. HLA-B∗07 and DRB1∗15 haplotypes are also more common in IgG4-SC [28]. Five single nucleotide polymorphisms are associated with the occurrence or higher activity of IgG4-SC: cytotoxic T-lymphocyte-associated protein 4 (CTLA4), tumor necrosis factor (TNF), Fc receptor-like 3 (FCRL3), trypsin 1 (PRSS1), and cystic fibrosis transmembrane conductance regulator (CFTR) [52–56]. Further analysis of genome-wide association studies will help determine the genetic risk for IgG4-SC.

The leading theory of the pathogenesis of IgG4-SC is an aberrant interaction between innate immunity, T-cell immunity, and B-cell immunity.

Activation of nucleotide-binding oligomerization domain-containing protein 2 (NOD-2) and toll-like receptor (TLR) on monocytes or basophils of IgG4-AIP patients activates the B-cell-activating factor (BAFF), leading to an enhancement of the IgG4 response. In an animal model, activation of TLR3 and TLR4 leads to immune-mediated cholangitis, pancreatitis, and sialoadenitis [57]. Macrophages, especially the M2 subtype, are abundant in bile duct tissue and can remodel the extracellular matrix of IgG4-SC patients [43]. The role of complement and the formation of circulating immunocomplexes in the pathogenesis of IgG4-RD are controversial. In IgG4-AIP, the level of complement is decreased and the level of circulating immunocomplexes in which IgG4 and its subtypes can be found is increased. However, the classical pathway through IgG1 appears to play a more important role in complement activation compared to the alternative pathway through IgG4 [58]. In patients with IgG4-SC, the Th2 lymphocytes response dominates with the release of IL-4, IL-5, and IL-13 [59]. The Th-2 cellular response generally leads to the maturation and proliferation of B cells and plasmacytes [57]. In IgG4-associated sialodacryoadenitis, IL-21 is released from both Th2 lymphocytes and Th follicular helper cells, leading to germinal center formation, but the incidence of these is lower in IgG4-SC than in IgG4-RD [43, 60, 61]. It should be noted that Th follicular helper cell levels correlate with both the number of plasmablasts and the serum IgG4 level [62]. These cells play an important role in the interaction of the T-cell and B-cell response in patients with IgG4-RD [43]. Tregs also play a fundamental role in the pathophysiology of IgG4-SC. Circulatory levels of naive CD45RA and Tregs are reduced, while levels of memory CD45RA-Tregs are increased [63]. Memory Tregs can inhibit the inflammatory response in the circulation and peripheral tissues, including bile tissue, while decreased levels of circulatory naive Tregs may lead to a multilocular inflammatory response, which may be pathogenic in patients with IgG4-RD [57]. In patients with IgG4-SC, high concentrations of FOXP3 + CD4 + CD25 + Tregs, which produce IL-10 and tumor growth factor-*β* (TGF-*β*), are present in the bile duct tissue. Costimulation of B cells with IL-4 and IL-10 increases IgG4 production [43]. On the other hand, TGF-*β* overproduction plays a very important role in the fibrogenesis of patients with IgG4-SC [64].

Chemokines play an important role in IgG4-RD. CCR8 expression was detected in half of Th2 lymphocytes and in 60% of FOXP3 Tregs [65]. CCR8-positive lymphocytes are present around bile ducts and peribiliary glands. CCL1 is also expressed in IgG4-SC in the ductal and glandular epithelia. Endothelial cells also express CCL1. The CCR8-CCL1 interaction may lead to obliterative phlebitis, which is a common pathological finding in IgG4-SC [66].

Patients with IgG4-RD have higher counts of plasmablasts and plasma cells but lower counts of CD19^+^ B-cells, CD20^+^ B-cells, and naive B-cells compared to the healthy population [67, 68]. IL-10-producing Bregs and circulating plasmablasts form IgG4 to an increased extent [43]. Remission of IgG4-RD on glucocorticoid treatment led to the depletion of naïve B-cells, plasmablasts, and plasma cells, while CD19^+^ B-cells and CD20^+^ B-cells were not altered. An increase of memory B-cells was observed only in patients who relapsed within two years of follow-up [68]. Compared to PSC, IgG4-SC has three activated immunological cascades (Fc-gamma receptor-mediated phagocytosis, B-cell receptor signaling pathway, and Fc-epsilon receptor I signaling pathway), and all three immunological pathways are associated with B cells or immunoglobulins, and conversely none of these pathways are directly linked to T cells in proteomic examination. These facts suggest a dominant role for B cells in the pathogenesis of IgG4-SC [43].

Okazaki et al. developed a pathogenic theory of IgG4-SC formation. So far, an imprecisely defined antigenic stimulus (self-antigen or microorganism) causes a decrease in naive Tregs, leading to the induction of a Th-immune response with the release of proinflammatory cytokines (interferon-*γ*, IL-1*β*, IL-2, and tumor necrosis factor-*α*). Subsequently, the Th-2 immune response is activated, leading to disease progression. An increased production of BAFF from monocytes and basophils and IL-10 from memory Tregs leads to increased production of IgG4, while upregulation of TGF-*β* from memory Tregs leads to fibrogenesis [57]. Cytotoxic T-lymphocytes play a crucial role in the pathogenesis of IgG4-RD. B cells present antigen and activate CD4+ cytotoxic T-lymphocytes; CD4+ cytotoxic lymphocytes dominate in immune cell infiltrate and decrease after targeted therapy [69]. Further studies will be needed to clarify the etiopathogenesis of this disease.

## 4. Classification

According to the 2019 American College of Rheumatology/European League Against Rheumatism classification criteria, a 3-step classification is recommended for determining a diagnosis of IgG4-RD. First, it must be demonstrated that a potential IgG4-RD case has the involvement of at least one of 11 possible organs in a manner consistent with IgG4-RD. Second, exclusion criteria consisting of a total of 32 clinical, serological, radiological, and pathological items must be applied. Third, eight weighted inclusion criteria domains, addressing clinical findings, serological results, radiological assessments, and pathological interpretations, are applied [70].

The classification of IgG4-SC is based on two clinical features: whether or not it is associated with AIP and the location of the bile duct stenosis. According to the association with AIP, we classify IgG4-SC into the following:

- The form associated with AIP
- IgG4-SC without AIP

**Figure 1:**
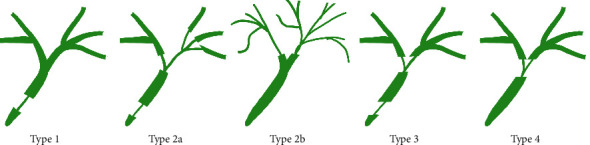
Schematic classification of IgG4-related sclerosing cholangitis by cholangiography.

The subtypes of IgG4-SC based on cholangiography appearance are the following (see Figure 1):

1. Type 1: the stenosis is located in the distal part of the bile duct. According to recent criteria, only the stenosis of bile duct in its intrapancreatic segment is classified as type 1; otherwise, it is type 4.
2. Type 2: Diffuse intrahepatic and extrahepatic stenoses are present. It has two subtypes: type 2a with prestenotic dilatations and type 2b without prestenotic dilatations with reduced bile duct branches caused by severe infiltration of plasma cells into the peripheral bile ducts.
3. Type 3: hilar stenosis + distal choledochal stenosis.
4. Type 4: isolated hilar stenosis (Figure 1).

Type 1 is mostly associated with AIP and should be distinguished from chronic pancreatitis, pancreatic cancer, and CC. Type 2 imitates PSC. MRCP is often sufficient to differentiate this type, and, in most of the cases, ERCP is not required for the diagnosis. A complementary test is liver biopsy, which verifies small bile duct involvement in PSC and colonoscopy, given the strong association of PSC and inflammatory bowel disease (IBD). Type 3 and type 4, which are associated with hilar stenosis, must be distinguished from CC. Endoscopic retrograde cholangiopancreatography (ERCP) with biliary tract biopsies, or endoscopic ultrasonography (EUS) and intraductal ultrasonography (IDUS), is necessary.

Approximately 90% of IgG4-SC patients have concomitant AIP [11, 29, 30]. The isolated IgG4-SC without AIP is less common, most often type 4. IgG4-SC without AIP type 1 has long been considered rare. Nakazawa et al. diagnosed 5 cases of isolated IgG-4-SC type 1, in three of which the diagnosis was made after surgery [15]. The observations of Naitoh et al. are interesting [13]. In a cohort of 872 patients with IgG4-SC, 62% of patients had type 1, 13.1% had type 2, 9.6% had type 3, 10.3% had type 4, and 3.9% had an unclassified type. Naitoh et al., like their predecessors, confirmed that the most common type of IgG4-SC is type 1, which accounts for more than 60% of patients with IgG4-SC [13, 29]. Naitoh et al. further compared the clinical features of patients with AIP-associated IgG4-SC and IgG4-SC without AIP. IgG4-SC without AIP was present in up to 16.3% (142/872) of IgG4-SC and was more common in women compared to the AIP-associated form. In IgG4-SC without AIP, the most common type was type 4 IgG4-SC, which was present in 30.9%, followed by type 1 in 23.8% of patients. This is the first large case series to report the distribution of stricture types in patients with isolated IgG4-SC. This work confirmed the conclusions from the past that the most common type of IgG4-SC without AIP is type 4 [16]. However, it pointed to a higher number of patients with IgG4-SC without AIP among patients with IgG4-SC and a higher number of patients with type 1 IgG4-SC among patients with IgG4-SC without AIP.

## 5. Diagnosis

  The diagnosis of IgG4-SC is based on a combination of four criteria:

1. A typical cholangiogram
2. Laboratory findings of elevated IgG4 antibodies
3. The presence of a systemic involvement
4. Histological examination

The aim is not only to determine the diagnosis of IgG4-SC as accurately as possible but also to exclude diseases with diametrically different treatment and prognosis, such as pancreatic cancer, PSC, and CC. Of the diagnostic methods, MRCP is sufficient in some cases; however, in most of the patients, ERCP with biliary tract biopsies, endoscopic ultrasonography-guided fine-needle aspiration of the pancreas or IDUS is required.

Currently, two diagnostic criteria are accepted in the diagnosis of IgG4-SC: the HISORt criteria, which were taken from the AIP diagnostic criteria and found their application mainly in Europe and USA, and the Japan Biliary Association criteria, which are used mainly in Japan and China. Both are based on a combination of the four diagnostic methods mentioned above. However, they differ in the definition of a certain and probable diagnosis of IgG4-SC.

In 2008, Ghazale et al. proposed applying the criteria originally created for the diagnosis of AIP to the diagnosis of IgG4-SC-HISORt [11]. Based on these, the diagnosis can be considered certain in a typical cholangiogram in combination with a laboratory or possibly histological finding. Diagnosis is probable if two of the following criteria are met: Criterion S, Criterion O, partially Criterion H, partially Criterion I. Patients with a probable diagnosis are indicated for trial treatment with corticoids. If they show a therapeutic response, the probable diagnosis can be reevaluated as definitive. Comparison between the HISORt criteria and the Japan Biliary Association criteria is summarized in Table 1.

In 2012, the Japan Biliary Association introduced diagnostic criteria that define a definite, probable, and possible diagnosis. They are listed in Table 2. Compared to the HISORt criteria, typical cholangiogram and elevated IgG4 do not mean a definitive diagnosis of IgG4-SC; only a possible diagnosis that needs to be verified by corticosteroid treatment. If the patient shows a response, the diagnosis should be reevaluated as probable. For a definite diagnosis, either a typical cholangiogram in combination with a precisely defined systemic involvement, a fully developed histological finding, or combination of partial histological findings, elevated serum IgG4 and cholangiogram is needed.

Thus, we can conclude that the HISORt criteria emphasize a typical cholangiogram; a typical histological finding is not necessary for the definitive diagnosis, while serological evidence of elevated IgG4 antibodies has the same diagnostic weight as the histological finding. Systemic impairment is only an additional criterion for a probable diagnosis. On the other hand, the Japanese criteria emphasize the role of histology and in some ways elevate the evidence of a well-defined organ involvement meeting the criteria for IgG4 disease above the elevated serum IgG4 alone.

New diagnostic criteria were published in 2021 by the Japan Biliary Association [71]. These follow the Japanese criteria from 2012 and take into account the Japanese Clinical Diagnostic Criteria for autoimmune pancreatitis from 2018 [72]. The following findings of IgG4-RD should be considered in the diagnosis:

- A typical pathological finding is localized subepithelially and leads to a thickening of the biliary tract wall even in sections that appear normal on the cholangiogram.
- Almost 90% of patients with IgG4-SC have AIP; therefore, it is necessary to classify IgG4-SC into IgG4-SC associated with AIP and isolated IgG4-SC from the beginning, as both forms have separate diagnostic criteria.
- It is also necessary to classify IgG4-SC according to the cholangiogram from the beginning. IgG4-SC types 1, 2, 3, and 4 differ in differential diagnosis.
- The following organ manifestations are diagnostically significant: sialoadenitis/dacryoadenitis, retroperitoneal fibrosis, and IgG4-related kidney disease.
- The importance of ruling out hepatobiliary malignancy was emphasized by adding “no neoplastic cells detected” to the histology criteria.
- Good prognosis is predicted by a good response to corticosteroids, and response to steroids has become a separate diagnostic criterion. Because some malignant lesions may also respond to steroids, cytological or histological examination is always recommended before using steroids. Steroid effectiveness should be assessed based on ERCP and/or MRCP imaging within two weeks after their administration.
- New imaging modalities, such as EUS and IDUS, are important in differential diagnosis.

The Japanese Clinical Diagnostic Criteria for IgG4-related sclerosing cholangitis are listed in Table 2. A significant limit to their application in daily practice will probably be their complexity. The scoring system developed by Moon et al. on a sample of 39 IgG4-SC patients and 76 PSC patients appears to be significantly simpler for daily practice. Based on age, other organ involvement, and beading on the cholangiogram, it reports excellent discrimination (area under the receiver operating curve 0.99) between IgG4-SC and PSC [73]. It is detailed in Table 3.

## 6. Clinical Picture

IgG4-SC often has a dramatic course but ultimately a good prognosis given a good response to corticosteroids and a comparable risk of developing malignancies as the general population.

The clinical picture is dominated by abdominal pain and jaundice, which is the most common symptom in patients with IgG4-SC. In the above-mentioned retrospective study of Tanaka et al., which evaluated 527 IgG4-SC patients from Japan, 35% of patients had jaundice [29]. Similarly, in an epidemiological study by the same authors from 2020, jaundice was the most common symptom in approximately 40% of patients [20]. The incidence of jaundice was significantly higher in the IgG4-SC-AIP(+) group (42.9%) than in the IgG4-SC-AIP(−) group (31.0%) (*p*=0.010) [13].

Jaundice was twice as common in IgG4-SC patients in the United States and the United Kingdom, where it occurred in 77% and 74% of patients, respectively [11, 30]. Sudden jaundice, weight loss, and older age at the time of diagnosis mimic hepatobiliary malignancy. A certain portion of patients with IgG4-SC or PSC are asymptomatic at the time of diagnosis; the others suffer from pruritus or fatigue. Paradoxically, approximately 37% of patients with IgG4-SC from a recent study by Tanaka et al. in 2020 were asymptomatic at the time of diagnosis [20].

Decompensated liver cirrhosis is rare in IgG4-SC. The most common manifestation in these patients is bleeding from the esophageal varices, which occurred in 0.9% of them [20].

Association with IBD is rare in IgG4-SC; it does not exceed 5% and is usually an intestinal manifestation of IgG4-RD. On the other hand, up to 90% of patients with IgG4-SC have concomitant AIP [11, 29, 30]. The incidence of AIP was slightly lower, 83.7%, in the last work of Tanaka et al. from 2020 [20]. The association of AIP with IgG4-SC is explained by identification of four phenotypes of IgG4-RD: pancreatobiliary, retroperitoneum/aortitis, head and neck limited, and Mikulicz systemic. Patients with the pancreatobiliary phenotype have the highest serum IgG4 and IgE levels and high prevalence of diabetes mellitus [74]. Occasional involvement of the pancreas in PSCs is mostly associated with azathioprine treatment.

AIP is the most common, but not the only, systemic manifestation of IgG4-RD in patients with IgG4-SC. Many of them have extrapancreatic manifestations of IgG4-RD, such as retroperitoneal fibrosis, sialodenitis and dacryoadenitis (Mikulicz disease), mediastinal lymphadenopathy, or renal involvement. Lung involvement is manifested with nodule creation, pulmonary fibrosis, or interstitial lung disease. Retro-orbital disease, aortic involvement, and neurological symptoms, such as progressive encephalitis and pituitary mass causing hypopituitarism, are rare. Based on recent data, approximately 26% of IgG4-SC patients had extrapancreatic manifestations, with a higher rate in those with concomitant AIP [13, 20]. IgG4-related organ disease (OOI) is also an important diagnostic criterion for patients with IgG4-SC without AIP, as up to 18.5% of these patients had some of the types of IgG4-related OOI included in the Japanese Clinical Diagnostic Criteria for AIP 2018 [13]. In 2017, Tanaka et al. showed that the most common extrapancreatic manifestation in IgG4-SC patients was sialadenitis or dacryoadenitis manifesting in symmetrical bilateral swelling in 15% of IgG4-SC patients, followed by retroperitoneal fibrosis in 7% [29]. Lung, aorta, and kidney involvements were each found in 1% of patients with IgG4-SC. These statistics were similar in a follow-up study in 2020 [20]. The results of Ghazale et al. are different, where up to 26% of patients had renal impairment, 9% retroperitoneal fibrosis, 6% sialadenitis or dacryoadenitis, and 4% lung involvement and mediastinal lymphadenopathy [11]. It is more difficult to interpret the UK study in this regard, as it assesses the incidence of extrapancreatic manifestations in a cohort of patients with AIP and IgG4-SC [30]. IgG4-related sialadenitis was found in 18%, renal infiltrates or masses in 9%, lung involvement in 6%, retroperitoneal fibrosis in 3%, ocular manifestations in 2%, and neurological sequelae in 2%. The results of this work also reflect recent Japanese diagnostic criteria that define the following diagnostically significant organ manifestations: sialoadenitis/dacryoadenitis, retroperitoneal fibrosis, and IgG4-related kidney disease.

The incidence of CC is low in patients with IgG4-SC and varies from 0.09% to 0.7% in individual studies. This stands in contrast to PSC patients who have a 160-fold higher risk of CC compared to the general population and lifetime prevalence of 5–10% [29, 75–78].

### 6.1. IgG4 in Serum

The IgG4 antibody is one of the four subclasses of immunoglobulin G. Normally, it accounts for less than 5% of the total IgG value. According to some authors, 74–88% of patients with IgG4-SC have elevated serum IgG4 levels (higher than the upper limit of normal value (ULN) of 1.35 g/L) [4, 11, 13, 29, 79]. Both the American and European Association for the Study of Liver Diseases practice guidelines on the diagnosis and management of PSC suggest measuring IgG4 in all patients with possible PSC to exclude IgG4-SC [80, 81].

Not only are IgG4 antibodies specific for IgG4-RD but also they are elevated in some patients with bronchial asthma, pemphigus, and atopic dermatitis. Elevated serum IgG4 levels are also present in some patients with PSC and in some patients with hepatobiliary malignancies, which complicates differential diagnosis. For PSCs, elevated serum IgG4 levels were present in 9–26% of patients [73, 82–91]. In CC, 8–14% of patients have elevated serum IgG4 levels, especially those who have CC in the field of PSC. On the other hand, about 10% of IGG4-SC patients with a typical histological finding have normal serum IgG4 levels [85, 92, 93].

These facts have led to the dilemma of whether the cutoff for IgG4 is set correctly, especially for the differential diagnosis of IgG4-SC from CC. The most problematic group in this respect appears to be patients with IgG4-SC types 3 and 4, without AIP, but it turns out that a cutoff of 1.35 g/L is not sufficient even to distinguish IgG4-SC from PSC. Boonstra et al. showed that the ULN cutoff for IgG4 (1.4 g/L) yields a sensitivity of 90% with a specificity of 85% for IgG4-SC. Increasing the cutoff level to 2 × ULN increased the specificity to 98%; however, it decreased the sensitivity of IgG4 to 70%. The highest specificity for IgG4-SC was achieved when applying the 4 × ULN (sIgG4 > 5.6 g/L) cutoff with a sensitivity of 42% [85].

Ohara et al. evaluated the cutoff values for IgG4 between each cholangiographic type of IgG4-SC and patients with other diagnoses: pancreatic carcinoma (PCa), PSC, and CC. The cutoff values were 1.19 g/dL for type 1 IgG4-SC versus PCa (sensitivity 90.2%, specificity 93.9%), 1.25 g/dL for type 2 IgG4-SC versus PSC (sensitivity 96.4%, specificity 87.6%), and 1.82 g/dL for types 3 and 4 IgG4-SC versus CC (sensitivity 85.7%, specificity 96.6%). Increasing the cutoff to 2.08 g/dL increased the sensitivity for IgG4-SC types 3 and 4 to 100% [92]. Differential diagnosis of IgG4-SC and CC was also addressed by Oseini et al. who compared sIgG4 levels in a test cohort of 126 patients with CC and 50 patients with IgG4-SC as well as in a validation cohort of 161 patients with CC and 47 patients with IgG4-SC and showed 100% sensitivity for IgG4-SC at a cutoff 4 × ULN (sIgG4 > 5.6 g/L) [93].

Therefore, other laboratory differential diagnostic markers were sought. Boonstra et al. used individual IgG subtypes for differential diagnosis. In patients with an sIgG4 >1.4 and <2.8 g/L, incorporating the IgG4/IgG1 ratio with a cutoff at 0.24 in the diagnostic algorithm significantly improved specificity and allows one to distinguish IgG4-SC from PSC [85].

Literature data on IgG4 levels in patients with IgG4-SC without AIP and IgG4-SC + AIP differ. While Nakazawa et al., Graham et al., and Takagi et al. have sporadically shown that patients with the isolated IgG4-SC form have lower serum IgG4 levels, in a recent study by Naitoh et al., patients with IgG4-SC associated with AIP had serum IgG4 levels that were comparable to those of patients with IgG4-SC without AIP [13, 15, 94, 95]. However, if patients with IgG4-SC and AIP have a different OOI at the same time, their serum IgG4 levels are significantly higher than those in patients with IgG4-SC and AIP without another organ impairment. When looking at individual IgG4-SC types according to the cholangiogram, the highest serum IgG4 values were found in type 4 patients, regardless of whether it was IgG4-SC + AIP or IgG4-SC without AIP [13].

Unlike PSC and CC, patients with IgG4-SC have elevated IgG4 levels not only in serum but also in bile. A cutoff of 113 mg/dl has 100% sensitivity and specificity for IgG4-SC [96].

### 6.2. Imaging Methods

Bile duct visualization is the key in the differential diagnosis of bile duct stenoses. As a rule, it reflects the basic morphological changes in the bile duct wall which accompany stenoses. In the case of IgG4-SC, there is diffuse subepithelial lymphoplasmacytic inflammation of the wall of both intra- and extrahepatic bile ducts with fibrosis, with a preserved epithelial layer [97]. This pathological correlate is manifested in the imaging method by two typical characteristics: segmental and long strictures with prestenotic dilation and diffuse thickening of the bile duct wall, which exceeds the extent of stenosis [13]. Of the imaging methods, computed tomography (CT), MRCP, and ERCP play a key role in the diagnosis of IgG4-SC as methods that are generally available. The cholangiogram is clearer with ERCP than with MRCP, and ERCP remains the gold standard in the differential diagnosis of bile duct stenoses. MRCP can replace ERCP in the diagnosis of some cases of IgG4-SC types 1 and 2, and it is important in assessing changes in the pancreatic duct with irregular narrowing of the main pancreatic duct signaling AIP. The quality of the examination is tied to MRI magnet strength and imaging sequences used. The use of 3 Tesla MRI scanners allows a detailed evaluation of the biliary tree comparable to ERCP. IDUS and EUS are of increasing importance, as they more accurately show the thickening of the bile duct wall. The limit for their use is poorer availability in routine clinical practice.

### 6.3. Cholangiogram

The most common findings on cholangiogram are segmental (>3 mm) and long (>10 mm) strictures with prestenotic dilatation and stricture of the distal common bile duct [13, 98, 99]. In contrast, stenoses in PSC are short (1-2 mm), mostly affecting both the intra- and extrahepatic bile ducts. Stenoses in PSC alternate with short normal sections, creating a typical beaded necklace image. In addition to band-like strictures, beaded and pruned-tree appearance and diverticulum-like formation are typical of PSCs [73, 98]. Nakazawa et al. noted segmental stricture and long stricture with prestenotic dilation in 100% (26/26) and 42% (11/26), respectively, of cases with IgG4-SC with AIP [98]. No patient with IgG4-SC with AIP was found to have a band-like stricture, beaded appearance, or diverticulum-like formation [98]. Nishino et al. retrospectively evaluated the cholangiogram in 24 patients with IgG4-SC with AIP and their conclusions were similar: 100% (24/24) of patients with IgG4-SC with AIP had segmental strictures, and a long stricture with prestenotic dilation was found in 12.5% (3/24) of patients. No patient with IgG4-SC with AIP was found to have a band-like stricture, beaded appearance, or diverticulum-like formation [99].

A long stricture and segmental stricture were the most common findings on the cholangiogram in patients with IgG4-SC without AIP. Naitoh et al. reported these findings in 65.1% of patients with IgG4-SC without AIP. On the other hand, these patients had a relatively high band-like stricture and pruned-tree appearance (11.4% and 4.1%) typical of PSCs, making it difficult to diagnose IgG4-SC without AIP [13].

### 6.4. Thickening of the Bile Duct Wall

The thickening of the bile duct wall in IgG4-SC is circular, usually extends beyond the extent of the stenosis, and has a smooth inner and outer edge. It is visible in CT scans and on MRCP but is more precisely diagnosed by IDUS and EUS, allowing reliable differential diagnosis with CC. Wall thickening in the stricture-free area was found to be significantly more common in patients with IgG4-SC on IDUS than on EUS (80.9% versus 73.8%; *p*=0.045) [13]. Naitoh et al. documented IDUS IgG4-SC properties in a cohort of 23 IgG4-SC patients with AIP. The control group consisted of 11 patients with CC. The circularly symmetrical wall thickness, smooth outer and inner edge, and homogeneous inner echo in the stricture were significantly higher in IgG4-SC than in CC (*p* < 0.01). An important diagnostic feature is the wall thickness in IgG4-SC in areas without stricture on the cholangiogram, which was significantly greater than that in the case of CC (*p* < 0.0001). This study provided evidence for the inclusion of type 1 IgG4-SC: it showed that, in most patients with IgG4-SC type 1 with AIP, stenosis is due to thickening of the inflamed wall (73% of patients) and not external compression by the inflamed pancreatic tissue [14].

The same authors in another paper showed that more than 50% of patients with IgG4-SC without AIP have wall thickening at a nonstricture region. This number is significantly lower than that in patients with IgG4-SC with AIP; however, wall thickening at a nonstricture region is useful for diagnosing IgG4-SC without AIP [13].

### 6.5. Histological Findings

The aim of the histological examination is not only to confirm IgG4-SC but also above all to rule out malignant stenosis. To exclude cancers, it is important to perform a transampullary bile duct biopsy and bile duct brushing cytology [71]. Immunohistochemistry for IgG and IgG4 is an essential part of the histological examination, which is usually not sufficient on its own to establish a definitive diagnosis.

IgG4-SC is characterized by the following histological findings:

- Marked lymphoplasmacytic infiltration
- Eosinophilic infiltration
- More than 10 IgG4-positive plasma cells per high-power microscopic field in biopsy and >50 per high-power field in resection specimens
- A high IgG4/IgG-positive cell ratio (>40%)
- Storiform fibrosis which often contains lymphocytes and plasma cells
- Obliterative phlebitis, in which the venous lumen is closed by inflammatory cells and fibrosis [28, 71]

The histological criteria are the same for patients with IgG4-SC with AIP and patients with IgG4-SC without AIP [94]. Bile duct tissue, liver tissue, and the ampulla of Vater can be examined. Naitoh et al. reported more than 10 IgG4-positive plasma cells per high-power microscopic field in 16.9% (56/331) of bile duct biopsies, 15.8% (9/58) of liver biopsies, and 36.8% (75/204) of ampullary biopsies in patients with IgG4-SC. Paradoxically, patients with IgG4-SC without AIP had more than 10 IgG4-positive plasma cells per high-power microscopic field more frequently than patients with AIP (29.6% versus 12.8%, *p* < 0.001) [13]. Marked lymphoplasmacytic infiltration and fibrosis were found in 32.9% (109/331) of patients with IgG4-SC. A total of 0.6% (2/331) of patients with IgG4-SC had storiform fibrosis, and no patient had any obliterative phlebitis in this study. In this work, the histological yield from bile duct tissue, including after immunostaining, was relatively low compared to the histological yield from the ampulla of Vater [13]. Other authors came to a similar conclusion [12]. We can explain this fact very easily, as lymphoplasmocyte inflammation is subepithelial and unevenly distributed in the bile ducts, and samples taken from the bile ducts are very small. Epithelial disruption and the presence of inflammatory infiltration in the epithelial layer suggest PSC. Storiform fibrosis, obliterative phlebitis, and high IgG4/IgG-positive ratio are detected almost exclusively by histological examination of bile duct resections, as opposed to biopsies. Kawakami et al. compared the biopsy yield from bile ducts and the ampulla of Vater and found that the criterion of more than 10 IgG4-positive cells/HPF was met in at least one biopsy in 72% (21/29) of patients, while 31% (9/29) met this criterion in both biopsies. This study was limited by a small cohort of patients with IgG4-SC with AIP [100].

Several studies have confirmed a typical histological finding in ampulla of Vater tissue in patients with IgG4-SC with AIP affecting the head of the pancreas [101, 102]. It appears that biopsy of ampulla of Vater and subsequent histological examination is also important in patients with IgG4-SC without AIP, as more than 10 IgG4-positive plasma cells/HPF were found in 23.8% of the IgG4-SC without AIP cases [13]. Papillary biopsy should not be performed separately, however, as papillary biopsy is considered a supplementary method in order to increase the yield of histological findings.

The utility of liver biopsy in the diagnosis of IgG4-SC is relatively low. Lesions of IgG4-SC may be observed in the biopsy specimen, but a fully developed histological finding is rare. An exception is the work of Deshpande et al. who found that 6/10 patients with IgG4-SC had more than 10 IgG4-positive plasma cells/HPF on a liver biopsy and 70% of IgG4-SC patients had intrahepatic biliary strictures. IgG4-SC patients presented higher portal and lobular inflammatory scores compared to PSC patients. Eosinophiles were found in portal-based fibroinflammatory nodules in 50% of IgG4-SC patients [103]. Patients with IgG4-SC have a significantly higher number of IgG4 plasma cells on a liver biopsy than patients with PSC and CC [99, 104]. Despite this, clinicians cannot overly rely on this criterion to make a definitive diagnosis of IgG4-SC. Like elevated serum IgG4 levels, not only is the presence of IgG4 plasma cells specific for IgG4-SC but also it can be detected in patients with PSC and CC [19]. Liver resections had >50 IgG4-positive cells/HPF in 9% of patients with CC. Liver explants had >50 IgG4-positive cells/HPF in 15.6% of patients with PSC [105, 106].

## 7. Treatment

The following are indications for treatment:

- Symptomatic patients
- Asymptomatic patients with cholestasis
- Patients with subclinical disease that can lead to severe or irreversible organ failure [107]

### 7.1. Corticosteroids

Corticoids are the first line in the treatment of IgG4-SC. The goal of treatment is to induce and maintain remission. This is defined as remission of symptoms, achievement of a biochemical response, decrease of IgG4 levels, and normalization of the cholangiogram. The indication for corticosteroid therapy is unquestionable, with the only exception being perhaps patients whose current health status contraindicates corticosteroid therapy (such as avascular necrosis of the hip, severe psychosis, etc.). Response to empiric corticosteroid treatment is an auxiliary diagnostic criterion. Corticosteroids also have a place in the treatment of disease relapse.

### 7.2. Remission Induction

The recommended initial dose of prednisone is 0.6–0.8 mg/kg body weight daily for 2–4 weeks, followed by a reduction of 5 mg/week over 2-3 months [19, 107]. This recommendation is based on the conclusions of the works of Japanese authors. Kamisawa et al. evaluated the effect of steroid therapy in 459 patients with AIP [108]. Most patients were treated with an initial dose of 30 mg and 40 mg of prednisolone daily. Remission on treatment was achieved in 98% of patients, and the time to remission was not significantly different statistically between patients treated with initial doses of 30 mg/d and 40 mg/d (*p*=0.401). Therefore, the question arises as to whether a lower initial dose should be used in patients at risk for diabetes mellitus. There is no work that specifically targets patients with IgG4-RD and diabetes mellitus. However, we can conclude that the initial dose of corticoids will affect the likelihood of disease relapse. This is confirmed by the findings of a study by Shirakashi et al. who showed using multivariate analysis of data from 152 IgG4-RD patients that patients treated with an initial dose of 0.4–0.69 mg/kg/day of prednisolone showed lower relapse rates than those treated with an initial dose of <0.39 or >0.7 mg/kg/day [109]. The effect of corticosteroid therapy is prompt in most patients, even dramatic, and minimizes the need for bile duct stenting. Some authors recommend the use of i.v. pulse corticoid therapy to achieve a faster response [110].

### 7.3. Maintaining Remission

The following issues are open questions in the management of IgG4-SC:

- The need for maintenance treatment
- Duration of maintenance treatment
- Maintenance dose
- Bile duct stenting before corticosteroid therapy
- The role of steroid-sparing therapy

The first three issues arise from two facts. On the one hand, the reduction and discontinuation of corticotherapy lead to a relapse of the disease in about 30% of those with IgG4-SC [107]. Risk factors for relapse are proximal biliary tract involvement, pancreatic involvement, and IgG4 levels above twice the upper limit of normal [11, 79, 95]. On the other hand, long-term treatment with corticoids exerts other effects, including osteoporosis, infections, or steroid diabetes mellitus. This fact is not negligible, because IgG4-SC affects middle-aged and older patients. More information on maintenance treatment can be drawn from the data on patients with AIP. Sah et al. consider maintenance treatment as not required in all patients with AIP [111]. It is only recommended for patients who have already had relapses or are more likely to relapse. In contrast, the need for maintenance treatment in patients with IgG4-SC is signaled by a study from Mayo Clinic, which concludes that, with early discontinuation of corticosteroid therapy after 11 weeks of treatment, 53% of patients relapse within three months [11].

Similar results were reported from a UK cohort in which 97% of patients with AIP, including IgG4-SC with AIP, responded to steroid treatment, but 50% relapsed after cessation of steroids and 2% during steroid therapy [30]. The Japanese authors emphasize that most patients with IgG4-RD require a prednisone dose of 5–10 mg daily to maintain remission [112]. In another study, the risk of relapse in patients with AIP was lowest at a dose of 5 mg/day of prednisone (26.1%); increasing the dose to 7.5 mg or 10 mg/day did not increase the likelihood of maintaining remission [113]. According to recent Japanese data, maintenance treatment in patients with IgG4-SC can be discontinued if remission persists for more than three years [19]. With this conclusion, the current European recommendation is that maintenance therapy with glucocorticoids should be considered only in multiorgan disease or those with a history of relapse [107].

### 7.4. Relapse Treatment

In case of relapse, reintroduction of corticosteroids is indicated, possibly in combination with other immunosuppressive treatments.

### 7.5. Other Immunosuppressions

Nonsteroidal immunosuppressants are currently a second-line treatment and are indicated in

- Corticosteroid-resistant patients
- Corticosteroid-dependent patients
- Those patients where corticosteroid treatment is limited due to adverse reactions
- The treatment of relapse

The combined use of nonsteroidal immunosuppressants with steroids to induce remission is a matter of debate. The authors of the International Recommendation for the Treatment of IgG4-RD did not find a uniform answer to this question either [114].

Rituximab has been shown to be effective in inducing, maintaining remission, and treating relapse in patients with IgG4-RD, including IgG4-SC. It is a monoclonal anti-CD20 antibody that induces B-cell depletion, which may play a key role in the treatment of IgG4-RD. The effect of rituximab in patients with IgG4-RD is described in case series and case reports; however, no randomized prospective clinical study has been performed yet.

The work of Ebbo et al. which evaluated the effect of rituximab in 33 patients with IgG4-RD in inducing and maintaining remission and treating relapse is valuable. There, 93.5% of patients achieved a clinical response to rituximab treatment, and 51.5% were able to discontinue corticoids. After discontinuation of rituximab, 41.9% of patients relapsed, whereas long-term rituximab treatment was associated with longer relapse-free survival (41 versus 21 months; *p*=0.02) [115]. Similar are the recent findings of other European authors that have confirmed the role of rituximab in the treatment of difficult-to-treat patients with IgG4-RD [116]. Carruthers et al. evaluated the efficacy of 1000 mg of rituximab in 30 patients with IgG4-SC who were not treated by corticosteroids. Disease response was seen in 97% of participants; 47% of patients were in complete remission at 6 months, and 12 patients at 12 months. Serum IgG4 level declined from 911 mg/dL (138–4780 mg/dL) to 422 mg/dL (56–2410 mg/dL) at month 6 (*p* < 0.05), though only 42% of patients achieved normal levels [117].

There are controversial views on the use of rituximab in the induction of remission as a first-line treatment in high-risk patients with high IgG4 levels and multiorgan involvement. It can be used as an off-label therapy in corticosteroid-resistant and corticosteroid-dependent patients, as well as corticosteroid-intolerant patients, or for the treatment of relapse. The recommended dose is 1 gram intravenously every 15 days. Maintenance therapy with rituximab is associated with longer relapse-free survival and may represent a novel treatment strategy, especially for difficult-to-treat patients with IgG4-SC. Difficult-to-treat patients should be identified on the basis of the IgG4-responder score, which is a reasonable index to assess disease activity [118]. The score includes clinical presentation, the number and severity of organ involvements, the presence of organ dysfunction, and the urgency of treatment. A recently published meta-analysis showed that therapy of IgG4-related pancreatobiliary disease with rituximab is associated with a high remission rate and a higher relapse rate in the presence of multiorgan involvement, while adverse effects were limited [119].

Azathioprine, 6-mercaptopurine, mycophenolate mofetil, and methotrexate are other second-line treatment options. Their effects in patients with IgG4-RD, including IgG4-SC, are poorly studied; again, available are data mainly in patients with AIP. A Mayo Clinic study evaluated the effect of steroid-sparing immunosuppression, azathioprine 2.0–2.5 mg/kg/day, 6-mercaptopurine 1 mg/kg/day, and mycophenolate 750–1000 mg b. d., in 76 patients with relapse of AIP. Relapse-free survival was similar in patients treated with combination therapy (steroids plus immunosuppressants) compared to patients treated with a steroid in monotherapy (*p*=0.23). In the follow-up, treatment with steroid-sparing immunosuppressants failed in 45% of patients. Some had a second relapse treated with rituximab. This treatment was successful in 83% of patients [120].

The work of Huggett et al. who evaluated the effect of azathioprine at a dose of 2 mg/kg/day as an add-on therapy to corticoids in the treatment of relapse in patients with AIP was more favorable for second-line treatment. Treatment was ineffective in 19% and azathioprine was not tolerated in 31.7% of patients. In these, mycophenolate 500 mg–1,000 mg b. d., methotrexate 15 mg weekly, or mercaptopurine 1 mg/kg/day was used as an alternative, and the treatment was well tolerated. At the end of follow-up, 58% of patients maintained remission with second-line treatment without corticoids [30].

### 7.6. Stenting before Corticotherapy

The initial effect of corticoids is very prompt, so most patients do not need bile duct stenting prior to corticotherapy [121, 122]. Exceptions are patients with severe jaundice and cholangitis.

## 8. Conclusion

IgG4 sclerosing cholangitis is an important part of differential diagnosis of bile duct stenosis. It is a disease with a very good prognosis if the diagnosis is made early. Despite increasing information and recently updated diagnostic criteria, the diagnosis is relatively difficult in routine clinical practice. Initial corticosteroid therapy is effective in inducing remission; maintenance treatment with corticosteroids reduces the likelihood of relapse but does not completely prevent it and is associated with a risk of side effects. Therefore, there is an unmet need for randomized prospective studies to evaluate new maintenance treatment strategies, including the use of steroid-sparing treatment regimens and B-cell reduction therapy to prevent relapse and long-term complications of this disease.

## Figures and Tables

**Table 1:** Comparison between the HISORt criteria and the Japan Biliary Association criteria [4, 11].

	HISORt criteria	Japan Biliary Association criteria
(1) Other organ involvement	Extrabiliary manifestations consistent with IgG4-RD, such as pancreas (focal pancreatic mass/enlargement without pancreatic duct dilatation, multiple pancreatic masses, focal pancreatic duct stricture without upstream dilatation, pancreatic atrophy); Retroperitoneal fibrosis; Kidney (single or multiple parenchymal low attenuation lesions: Round, wedge-shaped, or diffuse patchy); Salivary or lacrimal gland (enlargement)	Coexistence of autoimmune pancreatitis, or IgG4-related dacryoadenitis/sialadenitis, or IgG4-related retroperitoneal fibrosis

(2) Histology	Lymphoplasmacytic infiltrate with >10 IgG4+ cells per high-power field within and around bile ducts; Obliterative phlebitis; Storiform fibrosis	(a) Marked lymphocytic and plasmacyte infiltration and fibrosis
		(b) Infiltration of IgG4-positive plasma cells >10 IgG4-positive plasma cells/HPF
		(c) Storiform fibrosis, obliterative phlebitis

(3) Serology	Raised serum IgG4 levels (>1.35 g/L)	Elevated serum IgG4 concentrations (≥135 mg/dL)

(4) Imaging	Strictures of the biliary tree including intrahepatic ducts, proximal extrahepatic ducts, intrapancreatic ducts; fleeting and migrating biliary strictures	Diffuse or segmental narrowing of the intrahepatic and/or extrahepatic bile duct associated with the thickening of bile duct wall

(5) Response to steroids	Normalization of liver enzymes and at least partial stricture resolution after steroid treatment	Effectiveness of steroid therapy

Definite IgG4-SC	2 + 4, 3 + 4	1 + 4, 2a & b+3 + 4, 2a & b & c, 2a & b & d
Probable IgG4-SC	2 of the following: 1, 3, partial 2, partial 4	3 + 4+5
Possible IgG4-SC	N/A	3 + 4

**Table 2:** Revised criteria of the Japan Biliary Association [71].

I.	Narrowing of the intrahepatic and/or extrahepatic bile duct	(a) ERC
		(b) MRCP

II.	Thickening of the bile duct wall	(a) EUS/IDUS
		(b) CT/MRI/US

III.	Serological findings	Elevated serum IgG4 concentrations (≥135 mg/dL)

IV.	Pathological findings among (i)–(v) listed below	(a) (i), (ii), and (v) are observed
		(b) (v) is observed
		(c) All of (i), (ii), and (v) and either or both of (iii) or (iv) are observed

		(i) Marked lymphoplasmacytic infiltration and fibrosis
		(ii) More than 10 IgG4-positive plasma cells per high-power microscopic field
		(iii) Storiform fibrosis
		(iv) Obliterative phlebitis
		(v) No neoplastic cells identified

V.	Other organ involvement (OOI)	(a) Type 1 autoimmune pancreatitis
		(b) IgG4-related dacryoadenitis/sialadenitis, IgG4-related retroperitoneal fibrosis, IgG4-related kidney lesion

VI.	Effectiveness of steroid therapy	

Definite diagnosis IgG4-SC associated with AIP	Types 1, 2	Ia/b + IIa/b + III/VI
	Types 3, 4	Ia + IIa + IVb + III/VI

Definite diagnosis isolated IgG4-SC	Types 1, 2, 3, 4	Ia + IIa + III + IVa/VI

Probable diagnosis IgG4-SC associated with AIP	Types 1, 2 Types 3, 4	Ia/b + IIa/b
		Ia + IIa + IVb
		Ia/b + IIb + VI

Probable diagnosis isolated IgG4-SC	Types 1, 2, 3, 4	Ia + IIa + Iva
		Ia + IIa + III + IVb
		Ib + IIa + III + VI

Possible diagnosis IgG4-SC associated with AIP	Types 3, 4	Ia/b + IIa Ib + IIb + III

Possible diagnosis	Types 1, 2, 3, 4	Ia + IIa + III/Vb/VI
Isolated IgG4-SC		Ib + IIb + III + VI

**Table 3:** Scoring system for the differentiation of IgG4-SC and PSC [73].

Variable	Category	Points
Other organ involvement	Yes	3
	No	0
Beaded appearance	Yes	0
	No	2
Age	<30 years	0
	30–39 years	1
	40–49 years	2
	50–59 years	3
	>60 years	4

Total score	Diagnosis	
0–4	Probable PSC	
5–6	Indicating diagnostic steroid trial	
7–9	Probable IgG4-SC	

## Data Availability

Studies supporting this review are appropriately cited in the manuscript text and listed in the list of references.

## Abbreviations

AIP: Autoimmune pancreatitis
BAFF: B-cell-activating factor
Bregs: Regulatory B cells
CC: Cholangiocarcinoma
CFTR: Cystic fibrosis transmembrane conductance regulator
CT: Computed tomography
CTLA4: Cytotoxic T-lymphocyte-associated protein 4
ERCP: Endoscopic retrograde cholangiopancreatography
EUS: Endoscopic ultrasonography
FCRL3: Fc receptor-like 3
HPF: High-power field
IBD: Inflammatory bowel disease
ICD-10: International Statistical Classification of Diseases and Related Health Problems
IDUS: Intraductal ultrasonography
IgG: Immunoglobulin G
IgG4-RD: IgG4-related disease
IgG4-SC: IgG4-related sclerosing cholangitis
MRCP: Magnetic resonance cholangiopancreatography
NOD-2: Nucleotide-binding oligomerization domain-containing protein 2
PCa: Pancreatic carcinoma
PRSS1: Trypsin 1
PSC: Primary sclerosing cholangitis
sIg: Specific immunoglobulin
TGF-*β*: Tumor growth factor-*β*
TLR: Toll-like receptor
TNF: Tumor necrosis factor
Tregs: Regulatory T cells
ULN: Upper limit of normal value.
